# Genetic influences on macrophage function and lipid uptake in atherosclerosis

**DOI:** 10.1097/MOL.0000000000000986

**Published:** 2025-03-28

**Authors:** Chris A. O’Callaghan, Jiahao Jiang

**Affiliations:** a Centre for Human Genetics, Nuffield Department of Medicine, University of Oxford, Roosevelt Drive, Oxford, UK; b Broad Institute, Cambridge, Massachusetts, USA

**Keywords:** foam cells, lipid-associated macrophages., lipid-handling macrophages, macrophages, oxidized low-density lipoprotein

## Abstract

**Purpose of review:**

Macrophages can accumulate lipid droplets in their cytoplasm resulting in the foamy appearance seen in various diseases, especially atherosclerosis. This review assesses new insights from single cell analyses into the role of different human macrophage subpopulations and genetic risk in atherosclerotic disease.

**Recent findings:**

Single cell transcriptomic studies have identified TREM2hi foamy macrophages as a key population in both human and mouse atherosclerotic plaques. In addition, a TREM1hi/PLIN2hi population in human plaques has pro-inflammatory properties. Combined single cell transcriptomic and epigenetic multiomic profiling identified a population of CD52hi lipid-handling macrophages that are enriched for heritability of atherosclerotic disease. Molecular mechanisms have been identified linking gene-regulatory effects of disease-associated polymorphisms to the macrophage response to ox-LDL.

**Summary:**

Recent studies have used singe cell approaches to provide new insights into macrophage subsets, their interactions with lipid species and their role in mediating genetic influences on disease risk.

## INTRODUCTION

Macrophages are widely distributed throughout tissues and lipid-loaded macrophages arise in a range of disease states including atherosclerosis, obesity, metabolic dysfunction-associated steatotic liver disease (MASLD) and diabetic nephropathy [1,2]. These lipid-loaded macrophages are often referred to as ‘foam cells’ based on the ‘foamy’ appearance of their cytoplasm on microscopy.

There has been intensive investigation of the role of macrophages in atherosclerosis, where three robust observations underlie understanding of atherosclerotic cardiovascular disease (ASCVD). Firstly, a family history of ASCVD increases the risk of the disease indicating a genetic contribution. Secondly, robust epidemiological evidence demonstrates a clear relationship between blood cholesterol level and risk of disease. Thirdly, simple light microscopy of atherosclerotic lesions demonstrates large numbers of immune cells, including macrophages with a foamy appearance due to the presence of intracellular lipid droplets. The aim of this review is to provide an overview of recent work that explores how these three factors may interact in the pathogenesis of atherosclerosis. This understanding provides a foundation for developing rationale therapeutic approaches to both treatment and prevention of atherosclerotic cardiovascular disease. It also has implications for understanding other diseases in which macrophages are found to be loaded with lipid as foam cells. 

**Box 1:**
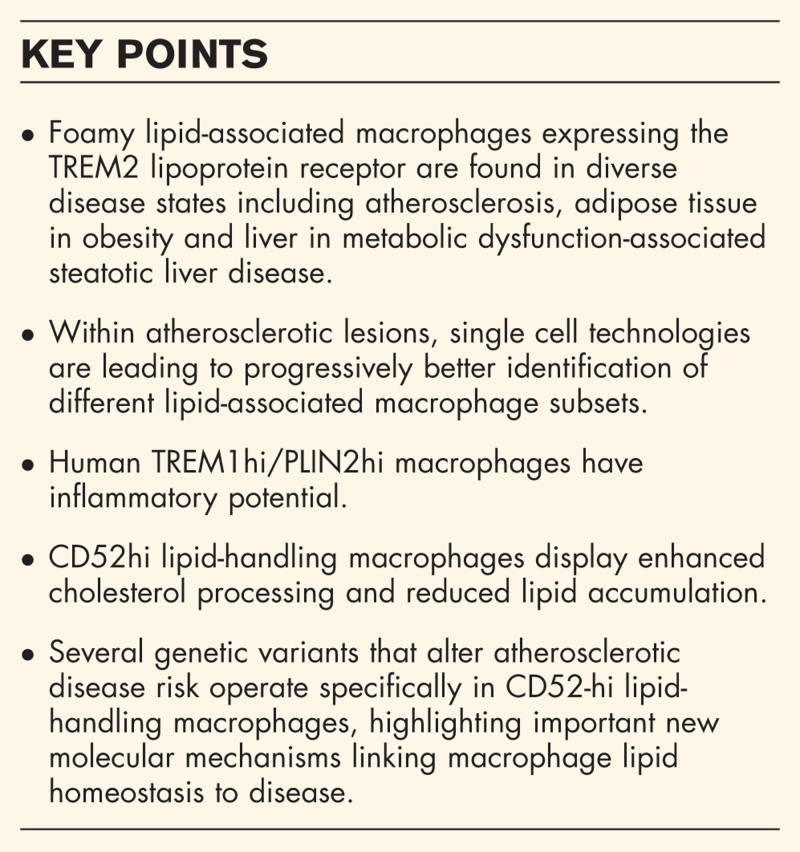
no caption available

## MACROPHAGE–LIPID INTERACTIONS

Early development of atherosclerotic lesions involves the entry of circulating lipoproteins into the arterial wall and their accumulation in the subendothelial space [3]. The presence of glycosylated molecules, such as proteoglycans and glycosaminoglycans favors modification of the lipoproteins, especially oxidation of low-density lipoprotein (LDL) to form oxidized-LDL (ox-LDL) [4]. Ox-LDL can be pro-inflammatory and promotes entry of immune cells into the vessel wall [5]. The cellular influx includes circulating monocytes which differentiate into macrophages within the vessel wall [6]. These macrophages have phagocytic activity and can take up modified lipoprotein, thus acquiring a foam cell appearance as the lipid accumulates as lipid droplets in the cytoplasm. These lipid-loaded macrophages undergo substantial changes in gene expression [7] and local release of this lipid load, through cell death or cholesterol efflux may trigger further influx of inflammatory cells and progression of the lesion.

## LIPID-ASSOCIATED MACROPHAGES

A variety of different types of macrophages have been identified with differing functional attributes as well as some plasticity in these attributes. In mouse the canonical types are M1 macrophages, which are broadly pro-inflammatory and M2 macrophages, which have broadly anti-inflammatory characteristics. In humans, this distinction has not been so clear. Adipose tissue in obesity and atherosclerotic lesions both contain lipid-loaded macrophages. Within obese adipose tissue there are large populations of macrophages and some macrophages become loaded with lipid to form ‘crown-like’ structures around large adipocytes [8]. In both atherosclerosis and obesity, lipid is stored in macrophages as lipid droplets which have an outer perimeter consisting of a monolayer of phospholipids. This monolayer incorporates multiple proteins including perilipin-2 (PLIN2) and other perilipin family members as well as enzymes relevant to lipid metabolism [9].

Studies of macrophages in adipose tissue have informed work on macrophages in atherosclerosis. Single cell RNA-seq was used to identify a subpopulation of macrophages that was present within adipose tissue, increased under obese conditions and conserved across mouse and humans [2]. These cells were derived from circulating monocytes and had a gene expression signature consistent with capacity for lipid metabolism and phagocytosis. A distinct feature was high expression of TREM2, a gene not expressed in other adipose tissue macrophages subpopulations. TREM2 encodes a cell surface receptor with an extracellular immunoglobulin variable domain and a positively charged amino acid in its transmembrane domain which allows it interact with DAP12 [10]. In this way, TREM2 is able to relay an activating signal through DAP12 which has an immunoreceptor tyrosine-based activation motif in its cytoplasmic domain. Ligands for TREM2 include phospholipids and lipoproteins [10].

The TREM2hi cells were termed lipid-associated macrophages (LAMs) and are the cells that form crown-like structures around adipocytes. LAMs also express genes involved in both lipid metabolism and phagocytosis – a combination that was not present in other adipose tissue immune cell types. In the presence of obesity, TREM2 deficiency reduced macrophage lipid accumulation, inhibited formation of crown-like structures and led to enhanced fat accumulation as well as raised levels of total cholesterol and LDL-cholesterol in blood [2]. A recent genome-wide CRISPR screen to identify genes that reduce ox-LDL uptake by macrophages demonstrated that TREM2 deficiency significantly impaired both ox-LDL uptake and the transition to a foamy phenotype [11^▪^].

## LIPID-ASSOCIATED MACROPHAGES IN ATHEROSCLEROSIS

The development of single cell approaches has allowed direct analysis of cells in both mouse and human atherosclerotic lesions and highlighted the presence of foamy TREM2hi LAMs. A meta-analysis of mouse single cell RNA-seq studies identified two subpopulations of Trem2hi macrophages, a Trem2hi-Slamf9 subpopulation and a Trem2hi-Gpnmb subpopulation [12]. The Trem2hi-Slamf9 population was enriched for transcripts such as Slamf9, Ch25h and Cd72 with immune potential, whereas the Trem2h-Gpnmb population was enriched for transcripts which are characteristic of foam cells and Trem2-response genes relevant to lipid metabolism, such as Lpl, Lipa, Fabp5, Apoc1, and Apoe. As Trem2hi-Gpnmb cells displayed a signature characteristic of the foamy phenotype and Trem2-response genes were increased in atherosclerotic lesions, the authors hypothesized that this population of cells arose in response to atherogenic conditions.

The precise effects of Trem2 deficiency on macrophage function in atherosclerosis in mouse models may be complex. A recent report demonstrated attenuation of atherosclerosis by Trem2 knockout in CX3CR1-expressing cells (which include monocytes and macrophages) on an Ldlr−/− background [11^▪^]. In this study, the mechanism appeared to be reduced uptake of lipid and so reduced formation of foam cells. Subsequently, the same group reported that the Trem2 agonist antibody AL002a increased macrophage survival and expanded plaque size [13]. Another group found that Trem2 deficient Ldlr−/− mice had increased necrotic core formation in early atherosclerosis [14^▪^]. The TREM2−/− macrophages had reduced expression of receptors for ox-LDL, reduced ox-LDL uptake and reduced survival in response to free cholesterol loading. The Trem2 agonist antibody 4D9, increased macrophage survival in response to free cholesterol loading and the authors concluded that TREM2 promotes macrophage survival.

Pooled data from two scRNA-seq studies of human atherosclerotic lesions [15,16], were used to identify three populations of macrophages which were termed hInflammatory-macrophages, hLYVE1-macrophages and hFoamy-macrophages, with the latter group having strong TREM2 expression and a signature including APOC1, APOE, FABP5, and FABP4 [12]. These human TREM2hi foamy macrophages had enrichment of marker genes characteristic of the mouse foamy Trem2hi macrophages (including TREM2, CD9, GPNMB, SPP1, CTSL, LIPA, and ACP5) and their gene expression patterns indicated enrichment of function related to the response to lipoproteins, lipid metabolism and lipid storage. Transcription factor analysis highlighted the role of NR1H3 in this human macrophage population and similar observations were also made in mouse foamy Trem2hi macrophages. Overall, there was significant conservation between human disease and mouse models of atherosclerosis. The transcriptional signature of the TREM2hi foam cells was similar to that of the equivalent LAMs seen in conditions including diet-induced obesity, metabolic dysfunction-associated steatotic liver disease and in neurodegenerative disease-associated macrophage-like microglia (DAM) [2,17,18].

## LIPID-ASSOCIATED MACROPHAGE SUBSETS AND INFLAMMATION

There are strong lines of evidence linking inflammation to atherosclerotic disease including the predictive value of inflammatory markers, such as C-reactive protein, interleukin-1-beta and interleukin-6, and trial data indicating benefit from anti-inflammatory therapy [19–21]. However, TREM2hi foamy macrophages appeared to have a transcriptional signature that was not notably pro-inflammatory. This issue was addressed using scRNA-seq of human carotid endarterectomy samples to identify 8 macrophage populations, including two LAM populations [22^▪^]. The two LAM populations were the previously reported TREM2hi population (expressing TREM2, CD9, GPNMB, SPP1, LIPA, and also CD36, FABP4 and FABP5) and a previously unreported population of TREM1hi/PLIN2hi macrophages. Both the TREM1hi/PLIN2hi and TREM2hi macrophage subpopulations express genes such as the lipid scavenger receptors CD36 and MARCO as well as the fatty acid-binding proteins FABP4 and FABP5. As previously described, the TREM2hi LAM have a transcriptional signature associated with lipid functions including lipid uptake, lysosomal metabolism, antioxidative function, cholesterol metabolism and cholesterol efflux. TREM2 LAM also have significant expression of the NR1H3 gene which encodes the transcription factor LXRa. However, the TREM1hi/PLIN2hi cells did not express genes involved in lysosomal degradation and cholesterol efflux, but did express genes associated with innate immune cell activation such as CCL2, CCL7 and IL1B. Trajectory analysis was consistent with the TREM1hi/PLIN2hi population arising from the TREM2hi population and some cells expressing both TREM1 and TREM2 were found in plaques. TREM1hi/PLIN2hi cells also expressed genes involved in apoptosis suggesting that they may have been undergoing apoptosis. TREM1hi/PLIN2hi macrophages have not been found in mouse models of atherosclerosis. The supernatant medium from cultured atheroma cells downregulated TREM2 and upregulated TREM1, PLIN2, CCL2, and IL1B in human monocyte-derived macrophages. TLR agonists also upregulated TREM1, but not TREM2, whereas ox-LDL upregulated TREM2 but not TREM1. Immunohistochemical studies confirmed that TREM2hi/TREM1negative LAMs were predominantly in superficial locations and adjacent to the fibrous cap whereas TREM1hi/TREM2negative LAMs were located deeper in the lipid core, close to lipid clefts. TREM1 and PLIN2 gene expression was greater in plaques from people who had recently had a stroke or transient ischemic event than in those with who had not. Taken together, these observations indicated that TREM1hi/PLIN2hi macrophages on a pathway towards apoptosis could contribute to inflammatory aspects of atherosclerosis and may have arisen from TREM2hi cells.

The combination of transcriptomic profiling and a panel of 274 antibodies using cellular indexing of transcriptomes and epitopes by sequencing (CITE-seq) has been used to study circulating monocytes in healthy volunteers and cellular populations in human carotid atherosclerotic plaques [23,24^▪^]. Multiple different monocyte subtypes were identified in healthy volunteers. The presence of a TREM1hi population was confirmed in carotid lesions. Two principle foamy macrophage sets were identified: a TREM2hi population expressing CD36 and genes involved in lipid metabolism such as ABCA1, LPL, and FABP5 and a further TREM2hi population expressing some foam cell genes such as APOE and inflammatory genes such as CCL18 and C1Q. Spatial transcriptomics has been applied to atherosclerotic lesions and demonstrates increased macrophages numbers in unstable plaques compared to stable plaques, but high resolution mapping of the location of different macrophages subtypes is not yet straightforward [25^▪^].

## LIPID-HANDLING MACROPHAGES AND GENETIC RISK OF ATHEROSCLEROTIC DISEASE

**FIGURE 1:**
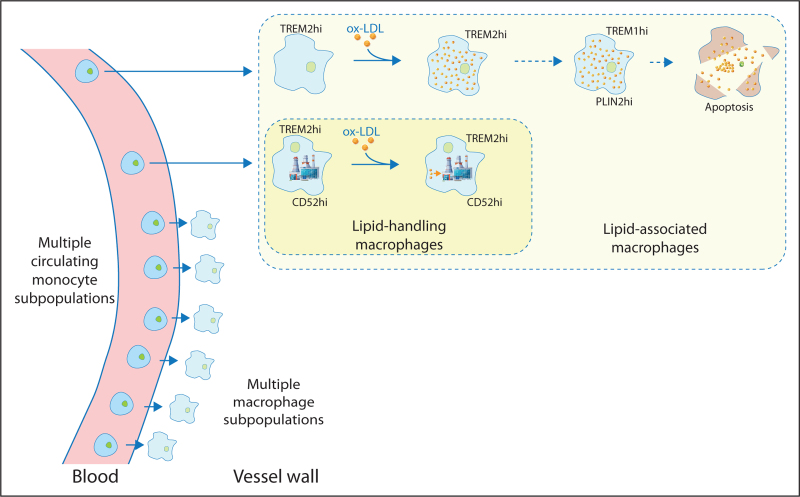
Lipid-associated macrophage subpopulations in atherosclerosis. Monocytes that enter the vessel wall can differentiate into different macrophage subpopulations. It remains unclear which circulating monocyte subpopulations give rise to which macrophage subpopulations. Different populations of TREM2hi lipid-associated macrophages (LAMs) have been identified. CD52hi lipid-handling macrophages have high expression of the ox-LDL receptor CD36, so would be expected to take up ox-LDL. However, they do not accumulate as much intracellular lipid as other TREM2hi LAMs and have high levels of expression of genes associated with lipid metabolism.

Advances in single cell technology now allow single cell multiomic profiling which provides not only a transcriptomic profile using RNA-seq, but also adds a detailed epigenetic profile of chromatin accessibility using ATAC-seq for each cell studied [26]. This single cell multiomic approach provides substantially more information than scRNA-seq for the clustering of cellular subpopulations. Single cell multiomics with combined RNA-seq and ATAC-seq was used to study the response of human macrophages derived from circulating monocytes to the atherogenic lipid ox-LDL. [27^▪^]. Using this approach, 9 macrophage subpopulations were identified. Whilst all subpopulations shared the TREM2-hi expression signatures to some extent, one subset showed high expression of lipid metabolism genes such as APOC1 and LPL even when not exposed to ox-LDL. The cells in this subpopulation were termed ‘lipid-handling macrophages’. These lipid-handling macrophages were characterized by expression of the cell surface glycoprotein CD52 (Fig. 1). A meta-analysis of the available single cell studies of human plaques allowed identification of a CD52hi subpopulation of LAMs in human atherosclerotic lesions. Notably, there is strong expression and motif usage for the transcription factor NR1H3 in CD52-hi macrophages.

CD36 operates as an ox-LDL receptor and is expressed at a higher level in lipid-handling macrophages than in other macrophage subpopulations. When macrophages are exposed to ox-LDL, lipid-handling macrophages upregulate CD36, but accumulate less lipid than other macrophage subpopulations. This suggests that they have more efficient lipid/cholesterol processing or clearance capacity that other macrophage subpopulations, which is consistent with their high expression of genes involved in lipoprotein processing and lipid metabolism.

The multiomic approach provides quantitative information about both level of expression of transcription factors in each cell and the chromatin accessibility of motifs across the genome to which these transcription factors may bind. Integrated analysis was applied to compare macrophages before and after exposure to ox-LDL and highlighted the role of specific AP-1 transcription factor family members and the NFE-2 family member BACH1. Multiomic data also allows the identification of cis-regulatory elements (CREs) involved in gene regulation. CREs were significantly enriched for the presence of genetic variants that genome-wide association studies have shown to be associated with coronary artery disease (CAD). This demonstrates that the response of macrophage to ox-LDL may be influenced by the genetic variation that is associated with altered disease risk. Integration of the single cell data with ChIP-seq, eQTL and Hi-C data provided a detailed map of the gene regulatory changes that arise in response to ox-LDL in human macrophages.

As macrophage subpopulations differed in their epigenetic profiles and in their gene expression, it was hypothesized that the genetic risk variants for CAD could operate differentially across different macrophage subpopulations. That is, different macrophage subpopulations would differ in the extent to which they are influenced by disease risk variants. Using the method of stratified linkage disequilibrium score regression, the enrichment of CREs for disease-associated genetic variants was assessed across the different macrophage subpopulations. Compared to other macrophage subpopulations, lipid-handling macrophages were significantly enriched for CAD heritability indicating their likely important role in disease. CAD heritability was also enriched in the gene program associated with the ox-LDL response in these lipid-handling macrophages. These findings demonstrate that lipid-handling macrophages make a greater contribution to the heritable component of CAD than other macrophage subpopulations and do this, at least in part, through their response to ox-LDL. Extension of this heritability analysis demonstrated that human plaque LAMs showed strong enrichment of CAD heritability [27^▪^].

Further inspection of lipid-handling macrophage-specific CREs led to several molecular explanations of the enriched disease heritability. A single nucleotide polymorphism (SNP) in the MITF gene which increases disease risk, disrupts a motif for the transcription factor TEAD1 and is associated with lower MITF expression. A disease-protective allele at a SNP in LPL disrupts a motif for the transcription factor NR1H3 which is expressed in lipid-handling macrophage. A disease-risk SNP decreases the expression of the FDX1 gene by reducing the enhancer activity of an AP-1/CEBPB-binding CRE. FDX1 is involved in mitochondrial function and reduced FDX1 expression could impair the ability of lipid-handling macrophages to process lipid beneficially. In most of these cases, the cellular context required for noncoding risk variants (e.g., open chromatin, expression of transcriptional regulators and target genes) is specifically met in lipid-handling macrophages, corroborating the conclusion that lipid-handling macrophages make a larger contribution to disease heritability than other macrophage subpopulations.

## CONCLUSION

The presence of macrophages with a foamy appearance due to cytoplasmic lipid droplets is characteristic of a number of disease states including atherosclerosis. The application of multimodal single cell technologies is leading to progressive improvements in the characterization of different macrophage populations and their interactions with lipid species. Integration of these results with GWAS results is providing insight into the causal genes, cell types and molecular mechanisms whereby genetic variation influences disease risk. Together, these advances are clearing the path for rational development of therapeutic or preventive treatments.

## Acknowledgements

*We are grateful to our colleagues for helpful discussions.*

### Financial support and sponsorship

*The author acknowledges support from the Novo Nordisk Foundation (NNF15CC0018346 and NNF0064142), the British Heart Foundation (RG/F/22/110085 and RG/F/23/110105)*

### Conflicts of interest

*There are no conflicts of interest.*

## REFERENCES AND RECOMMENDED READING

Papers of particular interest, published within the annual period of review, have been highlighted as:

▪ of special interest
▪▪ of outstanding interest
